# Synergizing One Health with osteopathic medicine: toward a unified health approach (‘One HealthOM’)

**DOI:** 10.1093/biosci/biaf123

**Published:** 2025-08-21

**Authors:** P Gunnar Brolinson, Alexis M Stoner, Fred Rawlins, H Dean Sutphin, Pawel Michalak

**Affiliations:** Edward Via College of Osteopathic Medicine, Blacksburg, Virginia, United States; Edward Via College of Osteopathic Medicine, Spartanburg, South Caarolina, United States; Edward Via College of Osteopathic Medicine, Blacksburg, Virginia, United States; Edward Via College of Osteopathic Medicine, Blacksburg, Virginia, United States; Edward Via College of Osteopathic Medicine, Monroe, Louisiana, United States; Center for One Health Research, Virginia–Maryland College of Veterinary Medicine, Blacksburg, Virginia, United States; Institute of Evolution, University of Haifa, Haifa, Israel

**Keywords:** One Health, osteopathic medicine, interdisciplinary collaboration, public health

## Abstract

The intersection of One Health—a collaborative, interdisciplinary approach addressing the interconnectedness of human, animal, and environmental health—and osteopathic medicine, which emphasizes holistic and preventive care, offers a promising but underdeveloped synergy for advancing public health. This perspective explores how doctors of osteopathic medicine (DOs), one of the fastest-growing segments of US healthcare professionals, can help operationalize One Health principles through education, research, and clinical engagement. Although osteopathic medical colleges have yet to fully institutionalize One Health frameworks, several emerging initiatives suggest a path forward. We highlight institutional examples that demonstrate how osteopathic training and philosophy can be leveraged to enhance disease prevention, biosurveillance, interdisciplinary research, and outreach in underserved communities. Rather than presenting a mature framework, this article offers a call to action: to more systematically integrate One Health into osteopathic medicine and to recognize the untapped potential of DOs as contributors to global health resilience.

In an era marked by unprecedented global health challenges—ranging from zoonotic disease outbreaks to environmental degradation and climate change—the necessity for integrative health approaches has never been more critical (Mwatondo et al. 2023). In particular, the COVID-19 pandemic has dramatically underlined the intricate interdependencies between human health, animal health, and environmental conditions, highlighting the severe repercussions of failing to adequately address threats emerging at their intersections (de Garine-Wichatitsky et al. 2020, Lefrançois et al. 2023). One Health, an initiative that underscores the interdependence of human, animal, and environmental health, provides a robust framework for addressing these multifaceted issues (Adisasmito et al. 2022). Concurrently, osteopathic medicine, with its holistic and patient-centered philosophy (Shannon and Teitelbaum 2009), aligns seamlessly with the One Health paradigm. We examine the convergence of One Health and osteopathic medicine in the United States, highlighting the potential benefits, opportunities for collaboration, and the implications for future health practices.

One Health is a transdisciplinary approach that recognizes the interconnectedness of human, animal, and environmental health (Zinsstag et al. 2011, Atlas 2013). It promotes community-oriented cooperation and collaborative efforts across various sectors—including public health, veterinary medicine, environmental science, and policymaking—to achieve optimal health outcomes. The One Health framework is particularly pertinent in addressing zoonotic diseases, antimicrobial resistance, food safety, and environmental sustainability. By fostering communication and cooperation across disciplines, One Health aims to create a unified response to complex health issues that transcend traditional boundaries (Lee and Brumme 2013).

Osteopathic medicine, established in the late nineteenth century by American physician and surgeon Andrew Taylor Still (1828–1917), emphasizes a holistic approach to patient care (Gevitz 2009). Doctors of osteopathic medicine (DOs, referred to as *DOs USA* in Australia) receive comprehensive training in conventional evidence-based medicine and surgery comparable to their MD counterparts. The curricula at DO-granting colleges of osteopathic medicine (COMs) are equivalent to those at MD-granting institutions, consisting of the first 2 years dedicated to biomedical and clinical sciences, followed by 2 years of core clinical training across various medical specialties (Freeman and Lischka 2009). In addition to this foundation, DOs are educated in the osteopathic principles that focus on treating the whole person, prioritizing preventive care, enhancing the body's innate ability to heal, and understanding the interrelationship between structure and function. Osteopathic practice may include hands-on manipulative techniques designed to improve patient outcomes, even though their use among DOs continues to decline (Healy et al. 2021). In the United States, osteopathic medicine is fully integrated into the healthcare system, with DOs practicing in all specialties and settings, ranging from primary care to surgical disciplines. As of 2024, DO students represented 25% of all medical students in the United States, reflecting the rapidly growing role of osteopathic medicine in shaping the future of healthcare (AACOM 2024).

We notice that the philosophical and practical tenets of One Health and osteopathic medicine are inherently complementary (table 1). Both frameworks advocate for a holistic understanding of health, emphasizing the importance of prevention and wellness and the interconnectedness of various health determinants (figure 1). However, One Health education and research initiatives within COMs remain scarce and are often in their early developmental stages, significantly lagging behind those established in veterinary schools and MD-granting institutions (Rabinowitz et al. 2017, Prozialeck and Edwards 2018).

**Figure 1.**
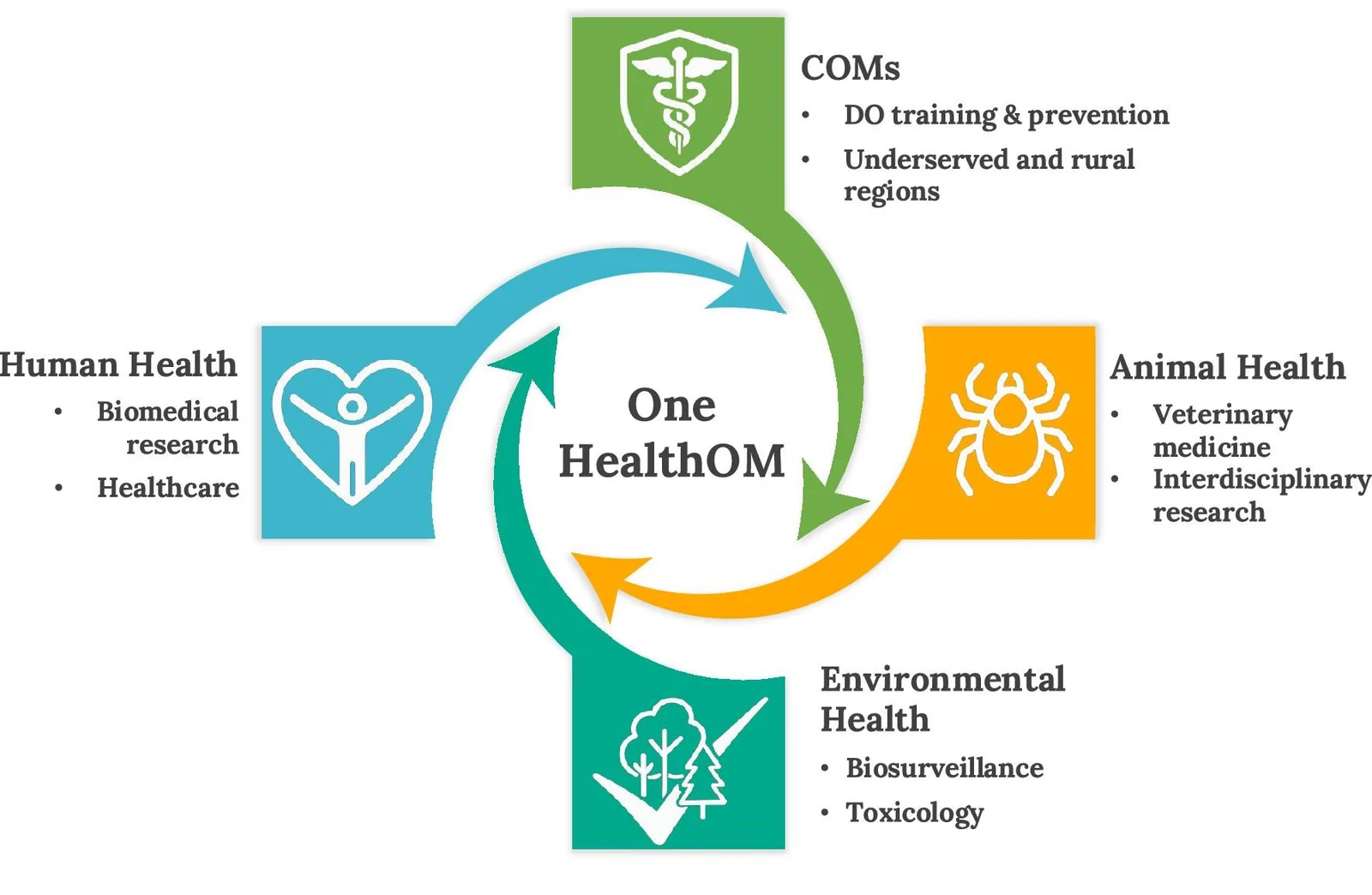
One HealthOM: Integration of osteopathic medical colleges (COMs) and osteopathic medical practice into the three main components of One Health conceptual framework.

**Table 1.** Conceptual alignment between One Health and osteopathic medicine.

**Aspect**	**Description**
Holistic approach	Osteopathic medicine's focus on the whole person mirrors One Health's integrative perspective on health determinants. Both disciplines encourage looking beyond immediate symptoms to understand underlying causes and contributing factors.
Preventive care	Preventive measures are central to both One Health and osteopathic practice. One Health's emphasis on preventing disease transmission between humans and animals and addressing environmental factors aligns with osteopathic medicine's focus on lifestyle interventions and early detection of health issues.
Interdisciplinary collaboration	One Health thrives on collaboration across disciplines, a principle that is echoed in the osteopathic approach to healthcare. Osteopathic physicians are trained to work within interdisciplinary teams, facilitating communication and cooperation that is essential for One Health initiatives.
Environmental considerations	Osteopathic medicine's recognition of environmental influences on health dovetails with One Health's focus on environmental sustainability as a component of public health. DOs can contribute valuable insights into how environmental factors affect patient health and community well-being.

*Note:* The table illustrates areas of philosophical and practical synergy between the two paradigms, including holistic care, preventive approaches, interdisciplinary collaboration, and attention to environmental determinants of health.

## Examples of One Health initiatives at colleges of osteopathic medicine

### Michigan State University college of osteopathic medicine: Mekong One Health Innovation Program

One example of One Health–centered activities within a COM is Michigan State University (MSU) College of Osteopathic Medicine's involvement in the Mekong One Health Innovation Program (Michigan State University 2024). Funded by the US Department of State's Bureau of East Asian and Pacific Affairs, this initiative addresses transnational health concerns in the Mekong River region—including emerging and zoonotic diseases, antimicrobial resistance, and environmental challenges. MSU osteopathic faculty collaborate with their counterparts in Lao People's Democratic Republic, Thailand, and Vietnam to promote the One Health approach in research, education, and policymaking. Through a series of short courses, research grants, and stakeholder engagement, the Mekong One Health Innovation Program aims to bolster regional capacity and cultivate a network of practitioners committed to addressing shared health threats in the lower Mekong subregion.

### Midwestern University/Chicago College of Osteopathic Medicine: A One Health approach to environmental toxicology

At Midwestern University/Chicago College of Osteopathic Medicine (MWU/CCOM), faculty in the Department of Pharmacology have harnessed the One Health Initiative as a strategic framework to expand research on environmental toxicology (Prozialeck and Edwards 2018). Over the past several years, MWU/CCOM investigators have studied how disruptions in the delicate balance of human, animal, and environmental health can adversely affect patients—for instance, by exploring how cadmium, polybrominated diphenyl ethers, and other toxicants affect both animals and people. This focus on environmental toxins has led to tangible gains in extramural funding, publications, and interdisciplinary collaborations, including partnerships with institutions such as Harvard University, Northwestern University, and local hospitals. Importantly, the integration of One Health principles at MWU/CCOM has also enriched the osteopathic curriculum by weaving environmental health topics into core pharmacology courses and offering research opportunities that consistently engage osteopathic medical students. The MWU/CCOM experience suggests that adopting the One Health Initiative can serve as a catalyst for both scientific discovery and student training at other COMs.

### Edward Via College of Osteopathic Medicine: One Health agenda

Edward Via College of Osteopathic Medicine (VCOM), with its campuses and clinical network spanning three climate zones in the United States, Central America, and the Caribbean (figure 2), emerges as a regional leader in next-generation pathogen detection solutions, response capacity, and proactive research agendas addressing pathogens with epidemic and pandemic potential. A compelling demonstration of an operational One Health framework unfolded during the COVID-19 pandemic, enabling a multitiered response, which encompassed early detection, basic and translational virology, as well as rural public health outreach.

**Figure 2.**
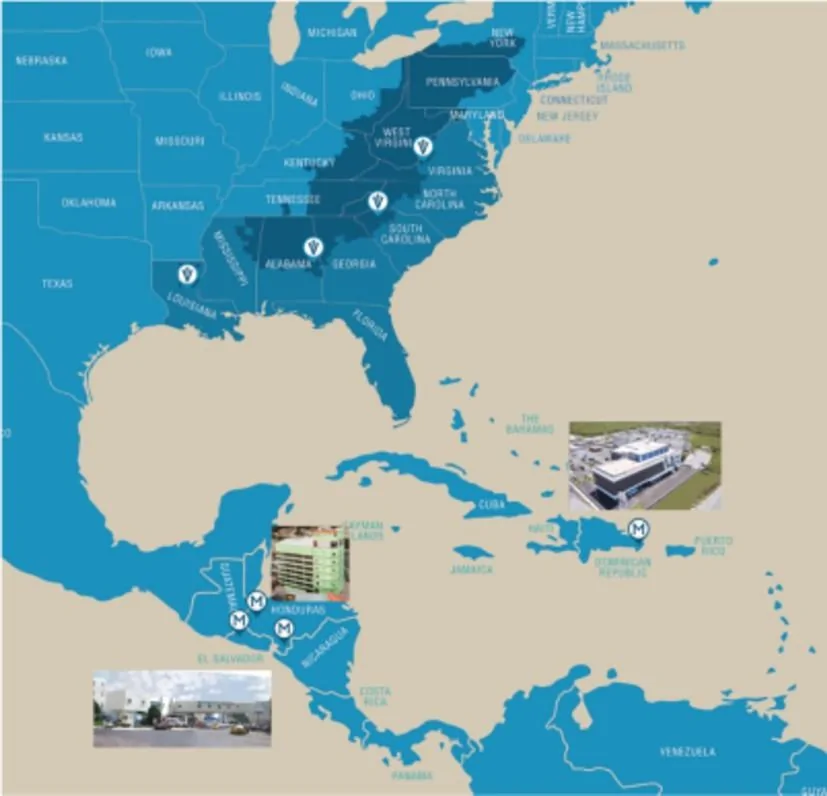
VCOM's Central America and Caribbean regional network of af!liated medical centers (marked as M).

At the detection level, VCOM collaborates with the Uniformed Services University of the Health Sciences (Bethesda, Maryland, in the United States) on the Syndromic Surveillance Collaborative Project, supported by a $5 million US Department of Defense earmark. This initiative leveraged the Via Learning Management System (VLMS), a web-based platform that logs clinical encounters from medical students during rotations. Having been operational for approximately 5 years, VLMS has accumulated over 4.8 million diagnostic and procedural entries, categorized using the World Health Organization's ICD-10 codes. This extensive data set enabled the successful benchmarking of VLMS in detecting early signs of the COVID-19 outbreak in the United States (Bruzda et al. 2021).

At the basic research level, One Health investigators at VCOM, in partnership with the Virginia–Maryland College of Veterinary Medicine (VMCVM), contributed to understanding SARS-CoV-2 emergence and evolution (Kang et al. 2021, Cereghino et al. 2024). For example, experimental research identified the spike A222V mutation as a potential adaptation to white-tailed deer, a known animal reservoir of the virus (Cereghino et al. 2025). These findings underscore the relevance of animal health in pandemic preparedness and affirm the critical need for cross-species viral evolution studies within a One Health framework.

The public health response extended to medically underserved rural populations. In partnership with the South Carolina Department of Health and Environmental Control, VCOM deployed a mobile medical unit to deliver COVID-19 and childhood vaccinations across school grounds and rural communities in Cherokee and Spartanburg Counties. Over 1700 vaccines were administered through this initiative, with medical students playing a major role in these outreach efforts, providing experiential learning while building trust and continuity of care in marginalized communities (Magalhaes et al. 2021, Skoczek et al. 2023). Together, these interlinked efforts, from syndromic surveillance to virological research to rural outreach, illustrate a functional One HealthOM approach in action, grounded in osteopathic principles and responsive to real-world public health threats.

In partnership with the VMCVM, VCOM has recently established the Center for One Health Research (COHR). This center brings together faculty, staff, and students from both institutions who share a common interest in cooperative research and scholarship focused on infectious diseases. The formation of COHR underscores a commitment to interdisciplinary collaboration, fostering an environment where expertise from both veterinary and osteopathic medicine converges to address complex health challenges. To support this initiative, significant renovations and remodeling of laboratories at the Virginia Tech campus have been undertaken to accommodate COHR's operations. These upgraded facilities are equipped with state-of-the-art technologies, enabling advanced research and facilitating collaboration among researchers. In addition, COHR has secured long-term seed funding designated for One Health collaborative research projects. This funding is intended to sustain and expand research endeavors that integrate human, animal, and environmental health perspectives, thereby advancing the One Health agenda through innovative and multidisciplinary approaches (figure 3). In addition to supporting studies on SARS-CoV-2, COHR's resources—including its biosafety level 3 laboratory and One Health Program funding—have been instrumental in enabling collaborative research on the emergence and evolution of mosquito- and tick-borne diseases (figure 3; Oakes et al. 2022, Cereghino et al. 2023). These efforts highlight COHR's role in advancing the One Health agenda through innovative, multidisciplinary approaches.

**Figure 3.**
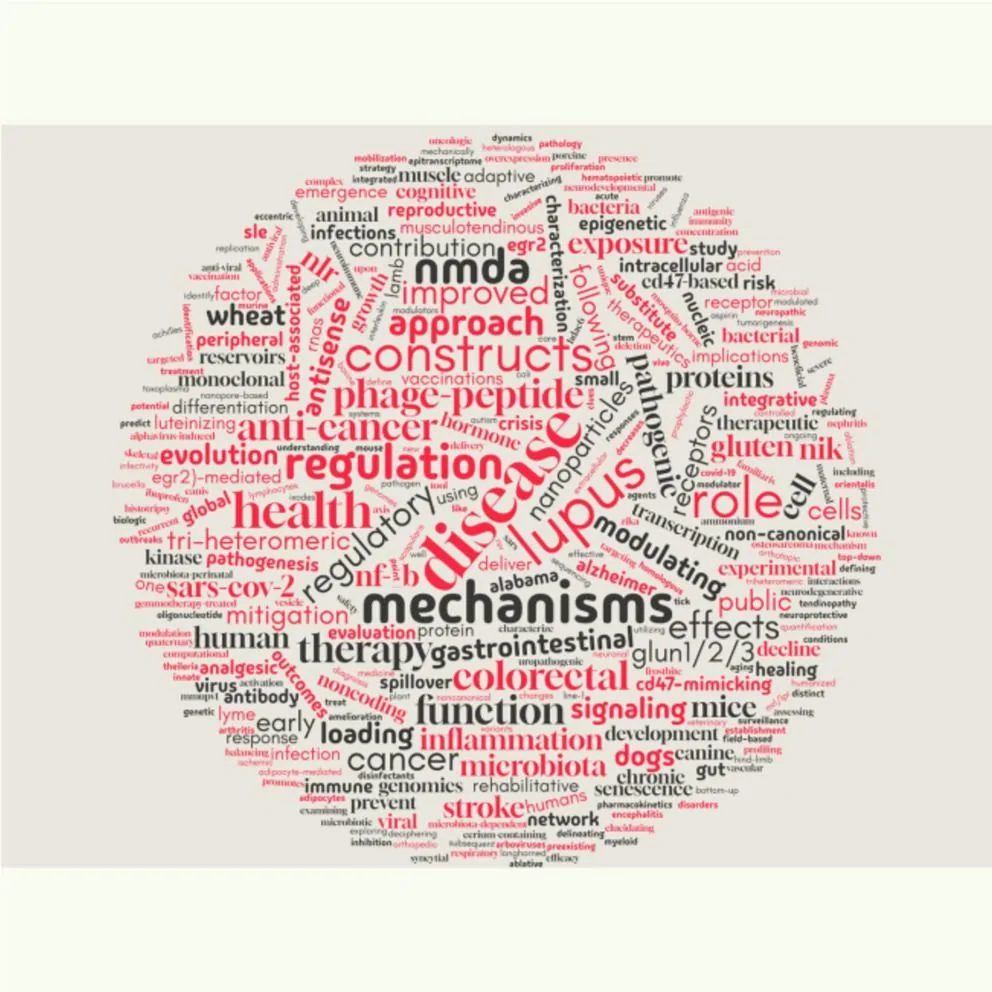
A word cloud depicting the titles of 66 research projects funded through VCOM's One Health Research Program (2016–2025), totaling US$2,983,840 in support. All projects in this program were required to meet defined One Health criteria and were conducted collaboratively between faculty at VCOM and the Virginia–Maryland College of Veterinary Medicine.

## Conclusions

Integrating One Health and osteopathic medicine may offer a transformative approach (One HealthOM) to advancing public health through holistic and interdisciplinary strategies, also aligning with the whole person health model, as was recently promoted by US Department of Veterans Affairs (Gaudet and Kligler 2019), the National Center for Complementary and Integrative Health in the National Institutes of Health (NCCIH 2021), and others. MSU/COM, MWU/CCOM, and VCOM exemplify this synergy by leading several One Health and global health initiatives. These efforts have enhanced disease surveillance and response capabilities, addressing challenges such as SARS-CoV-2 emergence and mosquito-borne diseases, while promoting sustainable health practices. Moving forward, expanding interdisciplinary partnerships, securing sustained funding, and embedding One Health principles into medical education will be essential for maximizing the impact of osteopathic medicine on global public health.

## Author Biography

P. Gunnar Brolinson, Fred Rawlins II, and H. Dean Sutphin are affiliated with the Edward Via College of Osteopathic Medicine, in Blacksburg, Virginia, in the United States. Alexis M. Stoner is affiliated with the Edward Via College of Osteopathic Medicine, in Spartanburg, South Caarolina, in the United States. Pawel Michalak (pmichalak@vcom.edu) is affiliated with the Edward Via College of Osteopathic Medicine, in Monroe, Louisiana, in the United States; with the Center for One Health Research at Virginia–Maryland College of Veterinary Medicine, in Blacksburg, Virginia, in the United States; and with the Institute of Evolution at the University of Haifa, in Haifa, Israel.
